# CIK细胞及其在肺癌治疗中的研究进展

**DOI:** 10.3779/j.issn.1009-3419.2011.12.10

**Published:** 2011-12-20

**Authors:** 虎 罗, 向东 周

**Affiliations:** 400038 重庆，第三军医大学西南医院呼吸内科 Department of Respiratory Medicine, Southwest Hospital, the Third Military Medical School, Chongqing 400038, China

**Keywords:** 肺肿瘤, CIK细胞, 过继免疫, Lung neoplasms, Cytokine-induced killer cells, Adoptive immunotherapy

## Abstract

肺癌作为恶性肿瘤导致死亡的首要原因, 严重影响人类的健康。由于细胞因子诱导的杀伤细胞(cytokine-induced killer cells, CIK)强大的增殖活性和细胞毒活性、非主要组织相容性复合体(major histocompatibility antigens, MHC)限制性以及低毒副作用等优点成为近年研究的热点。本文旨在介绍CIK细胞的基本特征及发挥作用的机制, 综述CIK细胞用于肺癌治疗的研究进展, 讨论CIK细胞大规模用于临床前需要解决的若干问题并作出展望。

## Abstract (en)

As the leading cause of cancer-related death among human beings, lung cancer seriously threats peoples' health all over the world.Cytokine-induced killer cells (CIK) is a new kind of adaptive immune cells characterized by its dramatic proliferation ability and cytotoxicity, non-major histocompatibility antigens (MHC) restriction and low side effects, which draw an increasing attention in recent years.The review is to introduce the basic features and mechanism of CIK cells briefly and summarize the researches of CIK cells used in the clinical therapy for lung cancer.Finally, we discuss several problems that should be addressed before CIK cells are widely applied to clinical patients.

肺癌尤其是小细胞肺癌(small cell lung cancer, SCLC)因其恶性程度高、预后差(5年生存率 < 5%), 已经成为各种癌症中导致死亡的首要原因^[1]^。由于手术切除的不彻底性、肺癌细胞对化疗的多重耐药性(multidrug resistance, MDR)和对放疗的低敏感性以及不可忽视的副作用等原因, 肺癌的复发、远处转移等难题长期不能得到有效解决。当传统治疗手段无法为肺癌患者提供满意的长期生存期, 新的治疗方法应运而生。

近年来基于免疫细胞在恶性肿瘤治疗方面的巨大潜力, 过继免疫作为生物治疗的有效手段之一已成为近年来肿瘤治疗的重要研究方向。到目前为止多种免疫细胞如树突状细胞(dentritic cells, DC)^[2, 3]^、细胞毒T淋巴细胞(cytotoxic T lymphocytes, CTL)^[4, 5]^、自然杀伤细胞(natural killer cells, NK)^[6]^、淋巴因子激活杀伤细胞(lymphokine-activated killer cells, LAK)^[7]^、抗CD3单克隆抗体诱导的杀伤细胞(anti-CD3 monoclonal antibody-induced killer cells, CD3AK)^[8]^、肿瘤浸润淋巴细胞(tumor-infiltrating lymphocytes, TIL)^[9]^等曾被用于肿瘤治疗的实验研究或临床治疗并显示出一定的抗癌能力。但由于体外扩增能力受限或安全性方面的问题, 这些免疫细胞在大规模临床试验研究中受到一定限制。而Schmidt-Wolf等^[10]^于1991年首次报道的细胞因子诱导的杀伤细胞(cytokine-induced killer cells, CIK)因其在体内外展现出来的强大抗肿瘤活性而备受关注, 迅速成为肿瘤免疫治疗的前沿和热点。

## 1 CIK细胞

研究^[11]^认为CIK细胞来源于CD3^+^CD56^-^T细胞, 是激活的Ⅱ型T淋巴细胞且同时兼具NK细胞的杀伤特性。但CIK细胞在外周血中含量甚微(约1%-5%)。因此要将CIK细胞用于临床治疗, 首先必须进行大量扩增。CIK细胞的产生过程大致如下:利用密度梯度离心法将患者或健康人外周血中单核细胞分离出来后, 通过添加外源性细胞因子如IFN-γ、IL-2、IL-1、OKT3(抗CD3的一种单克隆抗体)进行体外诱导扩增。体外扩增2周-4周后, 培养基中出现大量具有强抗癌活性的异质细胞群, 即为CIK细胞。CIK细胞主要包括CD3^+^CD56^+^T细胞、CD3^+^CD56^-^T细胞以及CD3-CD56^+^T细胞, 其中CD3^+^CD56^+^T细胞亚群被认为是发挥抗癌活性的主力军, 约占CIK细胞数量的40%-75%(也有文献将CD3^+^CD56^+^T定义为CIK细胞)。

各种外源性细胞因子的作用和机制并不相同, 如IL-2的作用机制可能是通过其受体共用链IL-2Rβ链和γ链, 经Jak/Stat信号转导通路参与细胞增殖, 同时促进NK细胞和T细胞定向分化为效应细胞发挥杀伤作用; OKT3的作用主要是刺激T细胞的增殖, 而对细胞毒活性影响不大; 而IFN-γ和IL-1对提高细胞毒活性非常重要, 并且不同的添加顺序对细胞毒活性影响甚大, 将IFN-γ添加在IL-2之前可以明显提高细胞毒活性, 其机制在于IFN-γ可以诱导IL-2受体(IL-2R)的高表达, 从而更有利于IL-2发挥作用。

## 2 CIK细胞基本特征

### 2.1 强大的体外扩增能力

大规模数量的杀伤活性细胞是过继免疫用于临床治疗的前提条件。LAK细胞在临床应用方面受到严重限制, 其主要原因在于其内在的弱增殖活性, 获得一定数量的细胞需要大量的IL-2, 因而造成的毒副作用尤其是毛细血管渗漏综合征不容忽视。CIK细胞不仅具有简单的体外扩增程序, 其同样具有强力的体外扩增能力。Jiang等^[12]^报道CIK细胞的增殖高峰出现在第21-28天, 细胞数量高达上千倍。

### 2.2 非主要组织相容性复合体(major histocompatibility antigens, MHC)非限制性的杀瘤活性

MHC限制性要求肿瘤细胞高表达某些信号分子以供自身抗原递呈细胞识别, 进而激活T细胞杀伤功能发挥抗肿瘤作用。但是近年研究证实, 肿瘤细胞的免疫逃避机制也是导致肿瘤治疗失败的主要原因之一。CIK细胞是最终效应细胞, 可以通过MHC非限制性途径^[13]^发挥对肿瘤细胞的直接杀伤作用, 而对正常细胞没有影响。

### 2.3 强大的细胞毒活性

CIK细胞的抗肿瘤活性主要依赖于CD3^+^CD56^+^T细胞的直接杀伤能力。Wang等^[14]^利用胶质瘤细胞系U87和U251比较CIK细胞和LAK细胞的抗癌能力, 研究证实CIK细胞拥有比LAK细胞更加强大的抗肿瘤能力。而Kim等^[15]^研究表明, 当CIK细胞与靶细胞比值为30:1时, 98%的NCI-H460肺癌细胞可被杀灭, 进一步证实了CIK细胞强大的细胞毒活性以及应用于临床的巨大潜力。

### 2.4 副作用少, 应用安全

免疫细胞应用于临床首先必须解决安全性问题, TIL细胞在临床应用方面受到限制, 主要是因为在细胞制备过程中, 肿瘤细胞的掺入有可能带来癌变的风险。到目前为止, 大量的动物实验和临床研究表明CIK细胞副反应少, 毒副作用低, 临床应用相对安全可靠。在一项40例病例参与的临床随机对照研究中, 研究者^[16]^利用脐血来源的CIK细胞治疗包括肺癌、胃癌、结肠癌等在内的多种实体瘤, 观察其副作用发现共有4例病例出现发热、流感样症状、乏力、肌痛症状, 对症处理后可以完全缓解。

## 3 CIK细胞作用机制

### 3.1 CIK细胞直接细胞毒活性

CIK细胞强大的抗癌能力主要归功于其CD3^+^CD56^+^T细胞亚群的直接细胞毒活性。这种直接杀伤机制可概括为:CIK细胞表面表达某些趋化因子或趋化因子受体, 可诱导其定向到达肿瘤细胞, 通过细胞表面的受体和肿瘤细胞表面的配体结合后, CIK细胞被激活。激活的CIK细胞释放穿孔素和细胞毒颗粒如α-氮-甲苯碳酰基-左旋-赖氨酸硫甲苯酯, 这些颗粒能够直接穿透封闭的靶细胞进行胞吐, 从而导致癌细胞的裂解, 但是整个过程不依赖于T细胞表面受体和Fas受体^[17]^。同时, 利用MHC Ⅰ类和Ⅱ类抗体不能阻滞CIK细胞对靶细胞的杀伤作用, 整个过程是非MHC限制的。研究^[10]^证实, 在CIK细胞与靶细胞的接触过程中利用淋巴因子功能相关抗原(lymphocyte function-associated antigen 1, LFA-1)或其配体细胞粘附分子(inter cellular adhesion molecule 1, ICAM-1)的单克隆抗体均可有效降低CIK细胞的杀伤活性。进一步研究发现, 在CIK细胞与靶细胞的接触过程中, CIK细胞表面的FcR使LFA-1和ICAM-1的结合状态从低亲和力转为高亲和力状态是其释放细胞毒颗粒的前提, 这些均表明细胞表面粘附分子在CIK细胞对肿瘤细胞的识别过程中起到重要作用。另外, 研究^[18]^认为CIK细胞表面分子如NKG2D、DNAM-1及NKp30等在CIK与靶分子的识别过程中起到重要作用。

### 3.2 分泌和免疫调节

CIK细胞兼具T细胞和NK的杀伤活性, 同时作为免疫细胞可以分泌多种炎性因子, 如IFN-γ、TNF-α、IL-2、IL-6和GM-CSF等, 直接或间接参与杀伤肿瘤细胞。Kornacker等^[19]^在用CIK细胞治疗慢性淋巴细胞白血病的研究中发现, CIK细胞分泌的IFN-γ能诱导慢性淋巴细胞白血病细胞上粘附分子ICAM-1的高表达, 进而提高CIK细胞诱导肿瘤细胞凋亡的能力。另外, CIK细胞可以与机体抗原递呈细胞如树突状细胞相互作用, 可提高树突状细胞的专职抗原递呈能力, 刺激机体CTL大量增殖, 从而大大刺激了机体对肿瘤细胞的反应性, 对于机体主动免疫应答起到很好的促进作用。

### 3.3 诱导肿瘤细胞凋亡或坏死

加速或诱导肿瘤细胞的凋亡或坏死也是近年来肿瘤治疗的重要研究方向。同时, Sun等^[20]^报道了CIK细胞可以诱导MGC-803胃癌细胞的凋亡并抑制其增殖能力, 免疫组化等方法证实CIK细胞杀伤胃癌细胞的机制为下调*p53*、*C-myc*和*Bcl-2*等基因的表达, 上调*Bax*基因的表达, 从而起到早期诱导胃癌细胞凋亡, 晚期诱导其坏死的效果。师岩等^[21]^研究证实, CIK细胞能诱导人肺癌细胞A549出现凋亡的超微结构变化, 流式细胞分析法检测早期凋亡率发现CIK实验组明显高于对照组, 提示体外培养的CIK细胞可明显诱导人肺癌细胞A549凋亡。

## 4 CIK细胞用于肺癌治疗的研究进展

近年随着过继免疫的迅速发展, CIK细胞也成为一种新的免疫细胞而倍受青睐。大量关于CIK细胞的动物实验和临床研究证实了CIK细胞用于肿瘤治疗的广阔前景。到目前为止, CIK细胞用于治疗肝癌、食管癌、乳腺癌、胃癌、结肠癌、肾细胞癌以及各种类型白血病^[22-25]^等取得初步成效, 在肺癌治疗方面也有新的进展。

### 4.1 CIK细胞治疗肺癌

Kim等^[15]^报道在体外实验中, 当CIK与肺癌细胞数比值为30:1时, 98%的NCI-H460肺癌细胞可被杀灭。在动物实验中, 当CIK细胞数为3×10^5^个和3×10^6^个时, 利用NCI-H460肺癌细胞接种产生的肿瘤体重可分别减轻57%和77%。李淑艳等^[26]^研究发现CIK细胞在体内外抑制Lewis肺癌细胞增殖, Fas/FasL途径在诱导肿瘤细胞凋亡中发挥一定作用, 同时CIK细胞抗肿瘤作用可能与淋巴细胞活化和分泌细胞因子有关。临床应用方面, Lin等^[27]^报道了1例伴发肺癌和类肿瘤皮肤病的多发性骨髓瘤病例, 患者在接受CIK细胞治疗后可有效控制肺癌进展。刘启亮等^[28]^和李文等^[29]^分别利用自体CIK细胞治疗晚期非小细胞肺癌(non-small cell lung cancer, NSCLC)患者取得初步疗效。其中, 后者疗效评价中总缓解率为78.1%, 临床症状评分改善率为86.4%-92.9%, 1年生存期达到80.4%。这些临床研究证据表明CIK细胞自体回输可能是治疗晚期肺癌良好过继免疫治疗方法, 可延长患者的生存期, 改善患者的生活状况, 且未见明显的毒副作用。

### 4.2 联合DC与CIK细胞治疗肺癌

CIK细胞在肿瘤治疗方面具有巨大潜力, 但是作为一种被动免疫治疗并不能长久地控制肿瘤的生长, CIK细胞联合DC疫苗成为主动免疫作为一种新的治疗策略开始涌现。DC是人体内主要的抗原递呈细胞, 其主要功能是诱导、调节及维持T细胞的免疫应答。另外, DC可以捕获肿瘤相关抗原, 表达淋巴细胞共刺激分子, 分泌各种细胞因子并启动细胞免疫和体液免疫。CIK细胞与DC共培养具有相互促进作用^[30]^, 可明显提高DC特异性的共刺激分子的表达和IL-12的分泌, 进而提高CIK细胞对肿瘤细胞的杀伤活性。体外实验中郑秋红等^[31]^和吕章春等^[32]^分别报道了肿瘤抗原致敏的DC诱导的CIK细胞对肺癌细胞的杀伤活性明显高于单纯的CIK细胞, 随着效靶比的升高, DC-CIK细胞对肺癌细胞的杀伤效应随之增强(*P* < 0.05), 并探讨DC增强CIK杀伤活性的可能机制为:DC表面的树枝状突起可使其负载肿瘤抗原并呈递给CIK细胞, 激活后的DC分泌IL-12、IL-18及IFN-γ等细胞因子, 刺激Th0、Th2细胞向Th1细胞分化, 并强烈激发Th1型特异性免疫应答。周永春等^[33]^利用DC疫苗联合CIK细胞治疗70例NSCLC的临床观察显示, DC-CIK治疗组中Ⅱ期、Ⅲ期患者的有效率分别为39.30%和28.60%, 与对照组的相应期别(Ⅱ期26.90%, Ⅲ期22.20%)相比差异有统计学意义(*P* < 0.05)。体内外实验均提示肿瘤抗原负载的DC细胞可增强CIK细胞对肺癌细胞的杀伤活性, 具有推广运用的价值。

### 4.3 联合化疗与CIK细胞治疗肺癌

尽管MDR的出现导致各种恶性肿瘤对化疗的反应不尽人意, 化疗仍然是多种肺癌尤其是SCLC患者的首选治疗方案。多项研究^[4, 34, 35]^证实, 化疗可以通过杀灭免疫抑制细胞(如髓系抑制性细胞、调节性T细胞)等方式提高机体对抗肿瘤的免疫应答, 增加肿瘤细胞对免疫细胞的敏感性。在一项59例晚期NSCLC患者参与的前瞻性临床研究中, Wu等^[36]^报道了与单独的化疗方案相比, 联合化疗和CIK细胞治疗晚期肺癌能明显提高患者机体免疫应答和生存质量, 其无进展生存期(*P*=0.042)和总生存期(*P*=0.029)均延长, 且无明显毒副作用。类似的, Niu等^[16]^将一线化疗失败的40例恶性肿瘤患者(包括15例肺癌患者)随机分为两组后分别予以二线化疗和联合二线化疗与CIK细胞治疗, 其无进展生存时间分别为2.03个月和3.45个月(*P*=0.031)。这些研究结果表明联合化疗与过继免疫可能作为一种新的治疗方案为肺癌患者带来更好的预后。

### 4.4 其他

鉴于CIK细胞在临床应用方面的巨大潜力, 各种用于提高CIK细胞增殖活性或杀伤活性的策略不断涌现。比如, 王士勇等^[37]^通过添加重组人纤维蛋白片段能明显增强CIK增殖活性, 但对CIK细胞的杀伤活性并无明显影响。多项研究^[38-40]^报道将DC疫苗、化疗和CIK细胞三者联合治疗肿瘤也取得一定成就, 可能成为未来肺癌治疗的新趋势。

## 5 CIK细胞存在的问题和未来的方向

尽管CIK细胞在体内外展现出的强大细胞毒活性等优势给临床肺癌治疗带来新的希望, 但在CIK细胞广泛应用于临床之前仍有许多问题丞待解决。

### 5.1 作用机制不够明确

到目前为止, CIK细胞发挥其强大细胞毒活性的机制还不够明确, 尤其是其与宿主免疫之间的复杂网络关系需要深入研究, 因为明确的作用机制才能为临床提供更好的理论基础和治疗靶点。如CIK细胞进入机体后, 其激活机制和发挥直接杀伤活性的机制有待进一步阐明。另外, 研究发现CIK细胞能够刺激机体T细胞大量增殖, 分泌各种细胞因子, 起到调整机体免疫的功能, 但是CIK细胞与机体免疫细胞之间的环路关系并不十分清楚。

### 5.2 CIK临床应用欠规范

科学、系统的制度和规范有助于CIK细胞临床研究的开展, 但到目前为止, CIK的制备和临床使用还没有形成统一的标准。如CIK细胞的数量和活力是保证临床疗效的关键, 但是从肺癌临床治疗的文献报道看, 各个研究单位使用的CIK细胞的制备方案和输注数量各有差异。另外, 不同单位对临床效果评价指标的选择也不尽相同, 由此导致CIK细胞的实际临床效果很难得到准确评估。CIK细胞用于肺癌临床治疗的报道主要是个案研究、前瞻性研究和少数的随机对照研究, 且多为短期疗效的评估; CIK细胞在肺癌临床治疗上的横向比较(肺癌与其他肿瘤的治疗效果对比)和纵向比较的(各种类型肺癌的治疗效果对比)资料还比较缺乏, 其对肿瘤强大杀伤作用和低毒副作用还没有足够的循证医学证据支持。因此, 大规模的临床随机对照研究或高质量的*meta*分析显得非常必要。

### 5.3 CIK的发展趋势和方向

如前所述, CIK细胞和DC疫苗、化疗联合应用时具有相互促进作用, 进而提高CIK细胞对肺癌细胞的杀伤活性。因此, 联合化疗、DC疫苗和CIK细胞作为一种新的治疗方案可能是未来肺癌乃至其他恶性肿瘤的发展方向, 但是, 联合治疗可能产生大量的过敏毒素和细胞因子^[41]^, 因此带来的副作用必须在应用之前得到更好的研究。另外, 体外实验^[16]^发现CIK细胞可以下调A549/CDDP和K562/ADR细胞系中耐药转运蛋白ABCG-2和P-gp的表达, 通过高表达CD3、CD56、FasL和CD59发挥对肿瘤耐药细胞的杀伤活性, 这无疑为以后耐药相关肿瘤的临床治疗提供了新的思路。最后, CIK细胞在其他疾病如慢性乙型肝炎^[42]^的治疗也见诸多报道, 但其治疗效果还有待进一步研究证实。

## 6 总结

肺癌作为癌症导致死亡的首要原因, 严重威胁着人类健康。传统治疗方案如手术、化疗等往往不尽如人意, 过继免疫作为一种新的治疗方法越来越受到重视, 而CIK细胞由于其强大的增殖活性和细胞毒活性、非MHC限制性以及低毒副作用等优点备受瞩目。但是, 在CIK细胞广泛应用于临床为肺癌患者服务之前, 还有诸多问题需要解决, 相信随着对CIK细胞的深入研究和更多循证医学证据的出现, 过继免疫治疗肺癌将会有新的突破。
